# 急性淋巴细胞白血病合并军团菌感染1例

**DOI:** 10.3760/cma.j.cn121090-20240823-00316

**Published:** 2025-01

**Authors:** 露晴 韩, 斌 张, 建 张, 捷思 李, 亚娟 刘, 亚肖 苏, 毅军 宋

**Affiliations:** 1 中国医学科学院血液病医院（中国医学科学院血液学研究所），血液与健康全国重点实验室，国家血液系统疾病临床医学研究中心，细胞生态海河实验室，天津 300020 State Key Laboratory of Experimental Hematology, National Clinical Research Center for Blood Diseases, Tianjin Key Laboratory of Cell Therapy for Blood Diseases, Haihe Laboratory of Cell Ecosystem, Institute of Hematology & Blood Diseases Hospital, Chinese Academy of Medical Sciences & Peking Union Medical College, Tianjin 300020, China; 2 天津医学健康研究院，天津 301600 Tianjin Institutes of Health Science, Tianjin 301600, China

患者，男，43岁，因“头痛、发热3周”于2023年11月13日入我院白血病诊疗中心。患者3周前开始无明显诱因下出现头痛、发热，体温最高39.1 °C，四肢酸痛，不伴畏寒、寒战，偶伴咳嗽、咳痰，痰呈白色黏液状，伴腹泻，就诊于当地医院，抗感染治疗效果不佳，骨髓细胞学检查发现骨髓增生减低，原始细胞占24％，急性白血病骨髓象，倾向于急性淋巴细胞白血病，遂转至我院。血常规：WBC 0.24×10^9^/L、RBC 3.75×10^12^/L、HGB 111 g/L、PLT 158×10^9^/L、ANC 0.01×10^9^/L；Na^+^115.36 mmol/L、Cl^-^ 84.28 mmol/L；ALT 177.09 U/L、AST 201.06 U/L、总胆红素（TBIL）34.63 µmol/L、直接胆红素（DBIL）11.50 µmol/L、间接胆红素（IBIL）23.13 µmol/L；肌酐（Cr）69.17 µmol/L；LDH 826.63 U/L。头部CT未见明显异常；胸部CT示：右肺中叶及两肺下叶间质病变（图1A）。骨髓细胞形态学及免疫分型提示为急性淋巴细胞白血病。染色体核型：46,XY,t(1;7)(q11;p11.1)[1]/46,XY[2]；融合基因检测：阴性；基因突变筛查：NRAS基因检测到p.G12V（16.30％，Ⅰ类）突变。诊断为急性淋巴细胞白血病，共予泼尼松3 d预治疗。于11月16日开始行R+VDCLP+VEN（利妥昔单抗+长春地辛+柔红霉素+环磷酰胺+培门冬酶+泼尼松+维奈克拉）方案化疗。化疗期间间断高热，体温最高达39.5 °C，新发畏寒、寒战，血培养提示铜绿假单胞菌，先后头孢他啶/舒巴坦、硫酸依替米星、亚胺培南、万古霉素及环丙沙星抗感染。化疗第5天体温未见明显改善，复查胸部CT示：双肺感染性病变，真菌感染可能性大，加用伏立康唑抗真菌，经验性加用利奈唑胺，试图覆盖可能的阳性菌感染。11月22日外周血二代测序（NGS）检出嗜肺军团菌（序列数41 251、物种相对丰富度99.61％、基因组覆盖度41.08％）、铜绿假单胞菌（序列数3）、黄曲霉（序列数2），考虑嗜肺军团菌为主要疑似病原，强化抗军团菌治疗，予阿奇霉素联合莫西沙星抗军团菌治疗。患者TBIL进行性升高达308.80 µmol/L，血氨33.00 µmol/L，周身皮肤及巩膜黄染，肝损加重，调整为卡泊芬净抗真菌治疗，并于保肝基础上联合血浆置换。2023年11月27日凌晨患者出现间断意识错乱，不配合治疗，自行拔出PICC导管，病情进展，暂停化疗，转入重症医学诊疗中心进一步治疗。

**图1:**
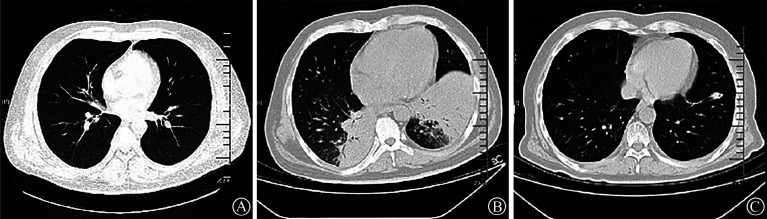
急性淋巴细胞白血病合并军团菌感染患者肺部CT表现 A 患者入院时胸部CT影像（右肺中叶及两下叶间质病变）；B 患者入重症监护病房（ICU）时胸部CT影像（两肺感染性病变较前进展，左侧胸腔积液）；C 患者出ICU后134 d胸部CT影像（两肺下叶感染性病变较前好转）

入科查体：体温38.0 °C，心率117次/min，呼吸频率33次/min，血压118/74 mmHg（1 mmHg＝0.133 kPa）。重度贫血貌，周身皮肤及巩膜重度黄染。双肺呼吸音粗，双肺下叶可闻及湿啰音。心律尚齐，各瓣膜听诊区未闻及额外心音及病理性杂音。腹软，肠鸣音活跃。四肢肌力可，肌张力不高。双下肢无水肿，双侧病理征阴性。血常规：WBC 7.71×10^9^/L，RBC 2.28×10^12^/L，HGB 66 g/L，PLT 77×10^9^/L，ANC 7.23×10^9^/L；C-反应蛋白（CRP）141.03 mg/L；Na^+^ 132.96 mmol/L，Cl^-^ 97.78 mmol/L；ALT 22.50 U/L，AST 23.48 U/L，TBIL 274.90 µmol/L，DBIL 168.90 µmol/L，IBIL 106.00 µmol/L。胸部CT示：双肺感染性病变较前进展（图1B）。头MRI平扫未见明显异常。初步诊断为急性淋巴细胞白血病、脓毒血症、意识错乱、高胆红素血症、重症肺炎、肠道感染等，不除外军团菌感染致全身系统受损表现。患者间断躁狂，拒绝治疗，拔管倾向明显，完善腰穿，脑脊液NGS检出嗜肺军团菌（序列数17），考虑军团菌为疑似病原，颅内军团菌感染诊断明确，继续阿奇霉素联合莫西沙星抗军团菌治疗，并加用依拉环素、泊沙康唑抗感染，保肝同时予床旁血液净化联合血浆置换治疗。追问病史，患者发病20多天前有公共浴池洗浴史。12月4日患者意识状态较前好转，偶伴意识混乱，无发热，复查胸部CT示：双肺感染较前好转，两下叶部分肺实变、不张，CRP降至24.58 mg/L。12月9日患者神志清楚，言语正常，但体温最高达38.8 °C，降钙素原（PCT）0.51 ng/ml，CRP上涨至85.15 mg/L，联用利福平3 d抗感染，体温逐步控制。12月18日患者体温正常，肺部感染较前好转，外周血NGS检出铜绿假单胞菌（序列数77），嗜肺军团菌（序列数18），考虑铜绿假单胞菌与嗜肺军团菌为疑似病原菌；脑脊液NGS阴性，停用莫西沙星及依拉环素，加用厄他培南短期抗感染，后复查胸部CT双肺炎症较前明显吸收，转回白血病诊疗中心治疗原发病。

讨论：军团菌感染在恶性血液病患者中虽然罕见，但具有严重的临床表现和较高的病死率，及时的诊断和有效的抗感染治疗是改善预后的关键。回忆整个诊治过程，患者发病初期细菌培养未见军团菌感染，积极采取mNGS进一步检测发现病原菌明确诊断，及时针对性治疗，最终临床症状缓解。在本例患者中，mNGS具有更高的检测敏感性，提供了更全面的微生物检测信息，起到了加快诊断的作用。

